# New optimum solutions of nonlinear fractional acoustic wave equations via optimal homotopy asymptotic method-2 (OHAM-2)

**DOI:** 10.1038/s41598-022-23644-5

**Published:** 2022-11-06

**Authors:** Laiq Zada, Rashid Nawaz, Wasim Jamshed, Rabha W. Ibrahim, El Sayed M. Tag El Din, Zehba Raizah, Ayesha Amjad

**Affiliations:** 1 Department of Mathematics, Abdul Wali Khan University Mardan, Mardan, Khyber Pakhtunkhwa 23200 Pakistan; 2 Department of Mathematics, Capital University of Science and Technology (CUST), Islamabad, 44000 Pakistan; 3 Mathematics Research Center, Department of Mathematics, Near East University, Near East Boulevard, PC: 99138 Nicosia/Mersin 10, Turkey; 4 Electrical Engineering, Faculty of Engineering and Technology, Future University in Egypt, New Cairo, 11835 Egypt; 5 Department of Mathematics, College of Science, King Khalid University, Abha, Saudi Arabia; 6 Faculty of Organization and Management, Silesian University of Technology, 44-100 Gliwice, Poland

**Keywords:** Mathematics and computing, Physics

## Abstract

The second iteration of the optimal homotopy asymptotic technique (OHAM-2) has been protracted to fractional order partial differential equations in this work for the first time (FPDEs). Without any transformation, the suggested approach can be used to solve fractional-order nonlinear Zakharov–Kuznetsov equations. The Caputo notion of the fractional-order derivative, whose values fall within the closed interval [0, 1], has been taken into consideration. The method's appeal is that it provides an approximate solution after just one iteration. The suggested method's numerical findings have been contrasted with those of the variational iteration method, residual power series method, and perturbation iteration method. Through tables and graphs, the proposed method's effectiveness and dependability are demonstrated.

## Introduction

Newly, there has been increasing attention to consuming fractional calculus to describe complex systems. Fractional derivatives are useful tools to model nonlinear phenomena since they allow us to capture the memory effects inherent in real systems. For example, the Riemann–Liouville derivative is widely used to model the evolution of viscoelastic materials. Fractional calculus also allows us to model the dynamics of complex systems that exhibit long-range interactions. In this regard, many researchers have well studied various schemes and aspects of partial differential equations (PDEs) and fractional order partial differential equations (FPDEs)^1–5^. However, recently much consideration has been paid to obtaining the solution of fractional models of physical concentration. Considering the views, the computational complexity involved in fractional order models is very crucial and it is difficult in solving these fractional models. Sometimes the exact analytical solution for each FPDE cannot be obtained using traditional schemes and methods. However, there are some schemes and methods that have proved efficient in obtaining an approximation to the solution of fractional problems. Among them, we draw the attention of readers to these methods and schemes^6–16^ which are used successfully. The Kerteweg de-Vries (KdV) equations play an important act in the application Zakharov-Kuznetsov (ZK) equations that analyzed the ionic-acoustic waves in magnetized plasma. It is an investigation of coastal waves in an ocean. The ZK equation was primarily found in the investigation of weak non-linear ion-acoustic waves in greatly attract losses plasma in two dimensions.

Recent works including the OHAM-2 are given by many researchers. Hashimet al.^17^ considered OHAM-2 for resolving numerous simulations of first-order fuzzy fractional IVPs. Olumide et al.^18^ studied the efficient result of the fractional-order SIR epidemic exemplary of childhood diseases with OHAM-2. Ahsanet al.^19^ presented a numerical result of a scheme of fuzzy fractional order Volterra integro-differential equation utilizing OHAM-2. Alshbool et al.^20^ assumed OHAM-2 to study the fractional Bernstein functioning matrices of Caputo types for resolving integro-differential equations. Hussain et al.^21^ employed OHAM-2 with special types of polynomials to join the system of Boussinesq equalities. Moreover, the HPM is utilized for many applications in fractional calculus. Peker and Cuha^22^ applied HPM in the Kashuri Fundo transform of fractional heat transfer and porous media equations. Abdul-Rahim et al.^23^ analyzed the fractional epidemic model via HPM. Qayyum et al.^24^ considered the method HPM as an application of arbitrary order film movement of the Johnson–Segalman liquid system. Dubey and Chakraverty^25^ presented an optimal solution for fractional wave equations by employing HPM. Chen and Liu^26^ used the local HPM for resolving coupled Sine–Gordon formulas in the fractal Domain.

In the present work, we investigated the following fractional ZK equation of the form,

$$D_{\tau }^{\alpha } F + \theta (F^{P} )_{\eta } + \psi (F^{Q} )_{\eta \eta \eta } + \rho (F^{R} )_{\eta yy} = 0,$$

where $F = F\left( {\eta ,y,\tau } \right),$ $\alpha$ is the parameter describing the construction of the fractional differential $(0 < \alpha \le 1)$, and $\theta ,\psi$ and $\rho$ are arbitrary parameters^7^. $P,Q,$ and $R$ are integers, responsible for the behavior of weak non-linear ion acoustic waves in a plasma containing cool ions and warm isothermal electrons in the being of a systematic magnetic field^27^.

The literature has utilized a variety of strategies to find both exact and approximative solutions to the ZK problem. One of these is the Perturbation Iteration Method (PIA) algorithm, which is used to solve the fractional order ZK problem in series^28^. For a fractional system of nonlinear ZK equations, Prakash et al. used the Sumudu transform approach and a new iterative strategy^29^. Eslami et al. examined the exact solutions to the modified ZK equation in^30^.

The second iteration of the optimal homotopy asymptotic mode was utilized in a similar way to establish a rough solution to the fractional order ZK equation. V. Marinca introduced the Optimal homotopy asymptotic method of the first version and second version namely called OHAM-1 and OHAM-II and used it for various differential equations in the series of papers^31–34^. Later, Liaqat Ali et al. used the suggested approach to solve a fluid mechanics-related differential equation^35^. The reason behind the organization of this research work is in the view of the above-mentioned literature:

- Fractional order nonlinear Zakharov–Kuznetsov equations are considered to study with the help of the second version of the optimal homotopy asymptotic method (OHAM-II) which is not explored yet in the available literature.
- The proposed method (OHAM-II) has never been used before for any type of fractional order model in the literature.
- The proposed method provides a series solution after only one iteration for the FZK equation which is the beauty of this method.
- According to numerical findings, OHAM-2 is the greatest at producing better and more accurate outcomes. It takes a few steps and leads to an almost precise result.

The remaining paper is organized as follows: In “Preliminaries" section, we introduce some basic definitions and properties of fractional Calculus. We will use them throughout the paper. In “Methodology" section, we give the theoretical foundation of the proposed method. In “Application of the OHAM-2” section, two examples are presented to illustrate the effectiveness of the proposed method. Finally, we complete the paper in “Conclusion" section by introducing the conclusion of our results.

## Preliminaries

In this portion of the research article, some fundamental meanings of fractional calculus, are presented. Like Riemann–Liouville, Grunwald Letnikov, Caputo, etc., which are related to our analysis.

### Definition 2.1

*R-L fractional integral*

$$I_{\eta }^{\alpha } g\left( \eta \right) = \left\{ {\begin{array}{*{20}l} {g\left( \eta \right)} \hfill & {{\text{if}}\,\,\alpha = 0} \hfill \\ {\frac{1}{\Gamma \left( \alpha \right)}\mathop \smallint \limits_{0}^{\eta } (\eta - \upsilon )^{\alpha - 1} g\left( \upsilon \right)d\upsilon } \hfill & {{\text{if}}\,\,\alpha > 0,} \hfill \\ \end{array} } \right.$$

hence ${\Gamma }$ denotes the gamma function defined as follows,

$$\Gamma \left( \omega \right) = \mathop \smallint \limits_{0}^{\infty } e^{ - \eta } \eta^{\omega - 1} d\eta \;\;\;\;\omega \in {\mathbb{C}},$$

### Definition 2.2

*The subsequent mathematical statement yields the Caputo operator of order for a fractional derivative*, *for* $n \in {\mathbb{N}}$, $\eta > 0$, $g \in {\mathbb{C}}_{\tau }$, $\tau \ge - 1$*.*

$$D^{\alpha } g\left( \eta \right) = \frac{{\partial^{\alpha } g\left( \eta \right)}}{{\partial \tau^{\alpha } }} = \left\{ {\begin{array}{*{20}l} {I^{n - \alpha } \left[ {\frac{{\partial^{\alpha } g\left( \eta \right)}}{{\partial \tau^{\alpha } }}} \right],} \hfill & {{\text{ifn}} - 1 < {\upalpha } \le {\text{n}},{\text{n}} \in {\mathbb{N}}} \hfill \\ {\frac{{\partial^{\alpha } g\left( \eta \right)}}{{\partial \tau^{\alpha } }},} \hfill & {} \hfill \\ \end{array} } \right.$$

### Lemma 2.3

*If* $n - 1 < \alpha \le n$ *with* $n \in {\mathbb{N}}$ *and* $g \in {\mathbb{C}}_{\tau }$ *with* $\tau \ge - 1,$ *then*

$$\begin{aligned} I^{\alpha } I^{\beta } g\left( \eta \right) & = I^{\alpha + \beta } g\left( \eta \right),\;\;\;\; \beta ,\alpha \ge 0. \\ I^{\alpha } \eta^{\lambda } & = \frac{{\Gamma \left( {\lambda + 1} \right)}}{{\Gamma \left( {\alpha + \lambda + 1} \right)}}\eta^{\alpha + \lambda } , \;\;\;\; \alpha > 0,\lambda > - 1, \eta > 0. \\ I^{\alpha } D^{\alpha } g\left( \eta \right) & = g\left( \eta \right) - \mathop \sum \limits_{k = 0}^{n - 1} g^{k} \left( {0^{ + } } \right)\frac{{\eta^{k} }}{k!}, \;\;\;\;{\text{for}} \,\,\eta > 0,n - 1 < \alpha \le n. \\ \end{aligned}$$

One can get more details regarding fractional derivatives in^13^.

## Methodology

In this section, the second version of the optimal homotopy asymptotic method has been protracted to fractional order PDEs. For this purpose, we consider the general nonlinear fractional order PDEs as,

1 $$\frac{{\partial^{\alpha } F\left( {\eta ,\tau } \right)}}{{\partial \tau^{\alpha } }} = {\text{A}}\left( {F\left( {\eta ,\tau } \right)} \right) + g\left( \eta \right) \alpha > 0,$$

Subject to I.C

2 $$\frac{{\partial^{\alpha - k} F\left( {\eta ,0} \right)}}{{\partial \tau^{\alpha - k} }} = h_{\kappa } \left( \eta \right). \left( {\kappa = 0,1,2,......,n - 1} \right), \frac{{\partial^{\alpha - n} F\left( {\eta ,0} \right)}}{{\partial \tau^{\alpha - n} }} = 0, n = \left[ \alpha \right].$$

3 $$\frac{{\partial^{k} F\left( {\eta ,0} \right)}}{{\partial \tau^{k} }} = g_{\kappa } \left( \eta \right). \;\;\;\;\left( {\kappa = 0,1,2,......,n - 1} \right),\;\;\;\; \frac{{\partial^{n} F\left( {\eta ,0} \right)}}{{\partial \tau^{k} }} = 0, \;\;\;\; n = \left[ \alpha \right].$$

$\frac{{\partial^{\alpha } }}{{\partial \tau^{\alpha } }}$ is the Caputo or Riemann–Liouville fractional derivative operator. $A$ is the differential operator and $g\left( {\eta ,\tau } \right)$ is the source term. The homotopy for (1) is, $\phi \left( {\eta ,\tau ;p} \right):{\Omega } \times \left[ {0,1} \right] \to R$

4 $$\left( {1 - p} \right)\left( {\frac{{\partial^{\alpha } \phi \left( {\eta ,\tau } \right)}}{{\partial \tau^{\alpha } }} - g\left( {\eta ,\tau } \right)} \right) - H\left( {\eta ,p} \right)\left( {\frac{{\partial^{\alpha } \phi \left( {\eta ,\tau } \right)}}{{\partial \tau^{\alpha } }} - ({\text{A}}\left( {\phi \left( {\eta ,\tau } \right)} \right) + g\left( {\eta ,\tau } \right)} \right) = 0,$$

In (4), the auxiliary function $H\left( {\eta ,\tau } \right)$ and embedding parameter p can be explored subsequently. We have added to Taylor's series about p by

5 $$\phi \left( {\eta ,\tau ,C_{i} } \right) = F_{0} \left( {\eta ,\tau } \right) + \mathop \sum \limits_{k = 1}^{m} F_{k} \left( {\eta ,\tau ,C_{i} } \right)p^{k} \;\;\;\; i = 1,2,3, \ldots$$

by Putting $p = 1$ in the above equation, we have

6 $$F\left( {\eta ,\tau ,C_{i} } \right) = F_{0} \left( {\eta ,\tau } \right) + \mathop \sum \limits_{k = 1}^{\infty } F_{k} \left( {\eta ,\tau ,C_{i} } \right) \;\;\;\; i = 1,2,3, \ldots$$

Putting Eq. (6) in Eq. (4) and comparing the co-efficient of the same powers of, $p$ and omit the remaining. Now the zero-order solution is obtained from the following,

7 $$p^{0} :\frac{{\partial^{\alpha } F_{0} \left( {\eta ,\tau } \right)}}{{\partial \tau^{\alpha } }} - g = 0,$$

and the first-order solution is obtained from (8)

8 $$p^{1} :\frac{{\partial^{\alpha } F_{1} \left( {\eta ,\tau ,C_{1} } \right)}}{{\partial \tau^{\alpha } }} = H\left( {\eta ,\tau ,C_{i} } \right)N\left( {F_{0} \left( {\eta ,\tau } \right)} \right),$$

Before applying $I^{\alpha }$ the above zero-order and first-order problems, firstly, we discuss the auxiliary function present in the first-order problem. The nonlinear operator is typically expressed as:

9 $$N\left( {F_{0} \left( {\eta ,\tau } \right)} \right) = \mathop \sum \limits_{i = 1}^{m} h_{i} \left( {\eta ,\tau } \right)g\left( \eta \right),$$

where $h_{i}$ and $g\left( \eta \right)$ and are known functions that are dependent upon the function $N$.

### Remark 3.1

*Where* $H\left( {\eta ,\tau ,C_{i} } \right)$ *random supplementary functions contingent on the initial approximation* $F_{0} \left( {\eta ,\tau } \right)$ *and a number of the unidentified parameters* $C_{i} ,i = 1,2,3..$*.*

### Remark 3.2

*The supplementary functions* $H\left( {\eta ,\tau ,C_{i} } \right)$ *is not unique and is of the same form like* $F_{0} \left( {\eta ,\tau } \right)$ *or the form of* $N\left( {F_{0} \left( {\eta ,\tau } \right)} \right)$ *or the combination of both* $F_{0} \left( {\eta ,\tau } \right)$ *and* $N\left( {F_{0} \left( {\eta ,\tau } \right)} \right)$*.*

### Remark 3.3

*If* $F_{0} \left( {\eta ,\tau } \right)$ *or* $N\left( {F_{0} \left( {\eta ,\tau } \right)} \right)$ *a polynomial function like* $H\left( {\eta ,\tau ,C_{i} } \right) = C_{1} \eta + C_{2} \eta^{2} ...$ *and if a trigonometric functions then* $C_{1} sinh\left( \beta \right) + C_{2} sinh\left( {2\beta } \right)...$*. If in special case* $N\left[ {F_{0} \left( {\eta ,\tau } \right)} \right] = 0$ *then it is an exact solution of * (1).

Ritz technique, association mode, Galerkins' technique, or least square process, by reducing the square residual error, can be used to determine the beliefs of unidentified parameters C_i_.

10 $$J\left( {C_{i} } \right) = \mathop \smallint \limits_{0}^{\tau } \mathop \smallint \limits_{\Omega } R^{2} \left( {\eta ,\tau ;C_{i} } \right)d\eta d\tau ,$$

where ${\mathcal{R}}$ is the residual assumed as follows,

11 $${\mathcal{R}}\left( {\eta ,\tau ,C_{i} } \right) = \frac{{\partial^{\alpha } \tilde{F}\left( {\eta ,\tau } \right)}}{{\partial \tau^{\alpha } }} - {\text{A}}\left( {\tilde{F}\left( {\eta ,\tau } \right)} \right) - g\left( \eta \right).$$

### Remark 3.4

*Our suggested approach is independent of any model's small or large parameters. Our effective tool has an auxiliary function that enables us to easily control and fine-tune the series solution's convergence after just one iteration.*

## Application of the OHAM-2

To expose the effectiveness and accuracy of the proposed method, we take the nonlinear high-dimensional FZK equations. For most of the computational work, we used the Mathematica 11 software package.

### Example 1

Consider the nonlinear fractional order FZK (3,3,3) equation in the following form,

12 $$\begin{array}{*{20}l} {\frac{{\partial^{\alpha } F\left( {\eta ,y,\tau } \right)}}{{\partial \tau^{\alpha } }} + \frac{{\partial F^{3} \left( {\eta ,y,\tau } \right)}}{\partial \eta } + 2\frac{{\partial^{3} F^{3} \left( {\eta ,y,\tau } \right)}}{{\partial \eta^{3} }} + 2\frac{{\partial^{3} F^{3} \left( {\eta ,y,\tau } \right)}}{{\partial \eta y^{2} }} = 0, \;\;\;\;0 < \alpha \le 1} \hfill \\ \end{array}$$

Subject to I.C

13 $$F\left( {\eta ,y,0} \right) = \frac{3}{2}\lambda {\text{sinh}}\left( {\frac{1}{6}\left( {\eta + y} \right)} \right),$$

where $\eta$ is an arbitrary constant.

14 $$\begin{aligned} L\left( {F\left( {\eta ,y,\tau } \right)} \right) & = \frac{{\partial^{\alpha } F\left( {\eta ,y,\tau } \right)}}{{\partial \tau^{\alpha } }}. \\ N\left( {F\left( {\eta ,y,\tau } \right)} \right) & = \frac{{\partial F^{3} \left( {\eta ,y,\tau } \right)}}{\partial \eta } + 2\frac{{\partial^{3} F^{3} \left( {\eta ,y,\tau } \right)}}{{\partial \eta^{3} }} + 2\frac{{\partial^{3} F^{3} \left( {\eta ,y,\tau } \right)}}{{\partial \eta y^{2} }}, \\ g\left( {\eta ,y,\tau } \right) & = 0, \\ \end{aligned}$$

Using OHAM-2 formulation, we get the $F_{0} \left( {\eta ,y,\tau } \right)$ from (7)

15 $$\frac{{\partial^{\alpha } F_{0} \left( {\eta ,y,\tau } \right)}}{{\partial \tau^{\alpha } }} = 0,$$

Apply the inverse operator $I^{\alpha }$ with initial condition both sides of (15), we have the following solution,

16 $$F_{0} \left( {\eta ,y,\tau } \right) = \frac{3}{2}\lambda {\text{sinh}}\left( {\frac{1}{6}\left( {\eta + y} \right)} \right).$$

By substituting (16) into (14), the nonlinear operator becomes,

17 $$N(F_{0} \left( {\eta ,y,\tau } \right) = \frac{3}{16}\lambda^{3} {\text{cosh}}\left( {\frac{\eta + y}{6}} \right)\left( {9{\text{cosh}}\left( {\frac{\eta + y}{3}} \right) - 7} \right).$$

The first approximation $F_{1} \left( {\eta ,y,\tau } \right)$ is given by (9),

18 $$\frac{{\partial^{\alpha } F_{1} \left( {\eta ,y,\tau } \right)}}{{\partial \tau^{\alpha } }} = H\left( {\eta ,y,\tau ,C_{j} } \right)N\left[ {F_{0} \left( {\eta ,y,\tau } \right)} \right].$$

The optimal supplementary function $H$ is selected in the custom

19 $$H\left( {\eta ,y,\tau ,C_{j} } \right) = {\text{C}}_{1} {\text{cosh}}\left( {\frac{\eta + y}{6}} \right) + {\text{C}}_{2} {\text{sinh}}\left( {\frac{\eta + y}{3}} \right) + {\text{C}}_{3} {\text{cosh}}\left( {\frac{\eta + y}{3}} \right) + {\text{sinh}}\left( {\frac{\eta + y}{6}} \right).$$

Using (17) and (19) into (18), with using $I^{\alpha }$ both side of (18) we get,

20 $$F_{1} \left( {\eta ,y,\tau } \right) = \frac{1}{{16\Gamma \left( {1 + \alpha } \right)}}\left[ {\begin{array}{*{20}l} {3\tau^{\alpha } \lambda \cosh \left( {\frac{\eta + y}{6}} \right)\left( { - 7 + 9\cosh \left( {\frac{\eta + y}{3}} \right)} \right)} \hfill \\ {\left( {\begin{array}{*{20}l} {C_{1} \cosh \left( {\frac{\eta + y}{6}} \right) + C_{3} \left( { - 7 + 9\cosh \left( {\frac{\eta + y}{3}} \right)} \right)} \hfill \\ {\sinh \left( {\frac{\eta + y}{6}} \right) + C_{2} \sinh \left( {\frac{\eta + y}{3}} \right).} \hfill \\ \end{array} } \right)} \hfill \\ \end{array} } \right]$$

By adding (16) and (20), we obtain the first order approximate result for FZK(3,3,3) by the following expression,

21 $$\tilde{F}\left( {\eta ,y,\tau } \right) = F_{0} \left( {\eta ,y,\tau } \right) + F_{1} \left( {\eta ,y,\tau ,C_{i} } \right).$$

With the domain $\sum { = \left[ {a,b} \right] = \left[ {0,1} \right]}$ the residual will be as

$${\mathcal{R}}\left( {\eta ,y,\tau } \right) = \frac{{\partial^{\alpha } \tilde{F}\left( {\eta ,y,\tau } \right)}}{{\partial \tau^{\alpha } }} + \frac{{\partial \tilde{F}^{3} \left( {\eta ,y,\tau } \right)}}{\partial \eta } + 2\frac{{\partial^{3} \tilde{F}^{3} \left( {\eta ,y,\tau } \right)}}{{\partial \eta^{3} }} + 2\frac{{\partial^{3} \tilde{F}^{3} \left( {\eta ,y,\tau } \right)}}{{\partial \eta y^{2} }}.$$

For finindg the $C_{i}$, we used the least sqarue method. Using the mathematical tenets of convergence control parameters from Table 1 and put in (21), we develop the first order approximate result for altered values of $\alpha$ for FZK (3,3,3).

**Table 1:** Auxiliary convergence control parameters for different values of $\alpha$ for FZK(3,3,3).

$\alpha$	$C_{1}$	$C_{2}$	$C_{3}$
0.5	− 0.34863102171153154	0.26590456732017476	− 0.7006972064317305
0.75	− 0.08716270059116164	0.291475794311555466	− 0.9634327396323357
1.0	− 0.18025964484745524	0.2824153038769191	− 0.8698875451125583

For $${\varvec{\alpha}} = 1.0$$

$$\begin{aligned} \tilde{F}\left( {\eta ,y,\tau } \right) & = \frac{3}{16}\lambda^{3} \tau \left( {9{\text{cosh}}\left( {\frac{{{\upeta } + y}}{3}} \right) - 7} \right){\text{cosh}}\left( {\frac{{{\upeta } + y}}{6}} \right) + \frac{3}{2}\lambda {\text{sinh}}\left( {\frac{{{\upeta } + y}}{6}} \right)0.282415{\text{sinh}}\left( {\frac{{{\upeta } + y}}{3}} \right) \\ & \,\,\,\, + {\text{sinh}}\left( {\frac{\eta + y}{6}} \right) - 0.869888{\text{cosh}}\left( {\frac{{{\upeta } + y}}{3}} \right) - 0.18026{\text{cosh}}\left( {\frac{{{\upeta } + y}}{6}} \right). \\ \end{aligned}$$

For $${\varvec{\alpha}} = 0.75$$

$$\begin{aligned} \tilde{F}\left( {\eta ,y,\tau } \right) & = 0.204012\lambda^{3} \tau^{0.75} \left( {9\cosh \left( {\frac{\eta + y}{3}} \right) - 7} \right)\cosh \left( {\frac{\eta + y}{6}} \right) + \frac{3}{2}\lambda \sinh \left( {\frac{\eta + y}{6}} \right) \\ &\left( {\sinh \left( {\frac{\eta + y}{6}} \right) - 0.963433\cosh \left( {\frac{\eta + y}{3}} \right) - 0.0871627\cosh \left( {\frac{\eta + y}{6}} \right)\left. { + 0.291476\sinh \left( {\frac{\eta + y}{3}} \right)} \right).} \right. \\ \end{aligned}$$

For $${\varvec{\alpha}} = 0.5$$

$$\begin{aligned} \tilde{F}\left( {\eta ,y,\tau } \right) & = 0.211571\lambda^{3} \tau^{0.5} \left( {9\cosh \left( {\frac{\eta + y}{3}} \right) - 7} \right)\cosh \left( {\frac{\eta + y}{6}} \right) + \frac{3}{2}\lambda \sinh \left( {\frac{\eta + y}{6}} \right) \\& \left( {\sinh \left( {\frac{\eta + y}{6}} \right) - 0.700697\cosh \left( {\frac{\eta + y}{3}} \right) - 0.348631\cosh \left( {\frac{\eta + y}{6}} \right)} \right.\left. { + 0.265905\sinh \left( {\frac{\eta + y}{3}} \right)} \right). \\ \end{aligned}$$

### Example 2

Deliberate the subsequent FZK (2,2,2) equation as

22 $$\frac{{\partial^{\alpha } F\left( {\eta ,y,\tau } \right)}}{{\partial \tau^{\alpha } }} + \frac{{\partial F^{2} \left( {\eta ,y,\tau } \right)}}{\partial \eta } + \frac{1}{8}\frac{{\partial^{3} F^{2} \left( {\eta ,y,\tau } \right)}}{{\partial \eta^{3} }} + \frac{1}{8}\frac{{\partial^{3} F^{2} \left( {\eta ,y,\tau } \right)}}{{\partial \eta y^{2} }} = 0,\;\;\;\; 0 < \alpha \le 1$$

with initial condition

23 $$F\left( {\eta ,y,0} \right) = \frac{4}{3}\lambda {\text{sinh}}^{2} \left( {\eta + y} \right),$$

where $\lambda$ is an arbitrary constant. For special case, when $\alpha = 1.0$ the exact solution for FZK(2,2,2) is

24 $$F\left( {\eta ,y,\tau } \right) = \frac{4}{3}\lambda \sinh^{2} \left( {\eta + y - \lambda \tau } \right).$$

The initial approximate $F_{0} \left( {\eta ,y,\tau } \right)$ is obtained from (7)

25 $$\frac{{\partial^{\alpha } F_{0} \left( {\eta ,y,\tau } \right)}}{{\partial \tau^{\alpha } }} = 0,$$

Apply the inverse operator, $I^{\alpha }$, with initial condition to (25), we have the following solution

26 $$F_{0} \left( {\eta ,y,\tau } \right) = \frac{4}{3}\lambda \sinh^{2} \left( {\eta + y} \right).$$

By substituting (26) into (22), the nonlinear operator becomes,

27 $$N(F_{0} \left( {\eta ,y,\tau } \right) = \frac{8}{9}\lambda^{2} \left( {5\sinh \left( {4\left( {\eta + y} \right)} \right) - 4\sinh \left( {2\left( {\eta + y} \right)} \right)} \right).$$

The first approximation $F_{1} \left( {\eta ,y,\tau } \right)$ is given by (10)

28 $$\frac{{\partial^{\alpha } F_{1} \left( {\eta ,y,\tau } \right)}}{{\partial \tau^{\alpha } }} = H\left( {\eta ,y,\tau ,C_{j} } \right)N\left[ {F_{0} \left( {\eta ,y,\tau } \right)} \right].$$

The optimal supplementary function $H$ is chosen in the form

29 $$H\left( {\eta ,y,\tau ,C_{j} } \right) = \frac{8}{9}\lambda^{2} \left( {C_{1} \sinh \left( {2\left( {\eta + y} \right)} \right) + C_{2} \lambda \sinh \left( {4\left( {\eta + y} \right)} \right) + C_{3} \lambda \sinh \left( {6\left( {\eta + y} \right)} \right)} \right).$$

using (27)and (29) into (28), with using $I^{\alpha }$ both side of (28) we get,

30 $$F_{1} \left( {\eta ,y,\tau } \right) = \frac{1}{{81\Gamma \left( {1 + \alpha } \right)}}\left[ {\begin{array}{*{20}l} {64\tau^{\alpha } \lambda^{4} \left( { - 4\sinh \left( {2\left( {\eta + y} \right)} \right) + 5\sinh \left( {4\left( {\eta + y} \right)} \right)} \right)} \hfill \\ {\left( {C_{1} \sinh \left( {2\left( {\eta + y} \right)} \right) + C_{2} \lambda \sinh \left( {4\left( {\eta + y} \right)} \right) + C_{3} \lambda \sinh \left( {6\left( {\eta + y} \right)} \right)} \right).} \hfill \\ \end{array} } \right]$$

By adding (26) and (31), we obtain the first order approximate result for FZK(2,2,2) by the succeeding appearance,

31 $$\tilde{F}\left( {\eta ,y,\tau } \right) = F_{0} \left( {\eta ,y,\tau } \right) + F_{1} \left( {\eta ,y,\tau ,C_{i} } \right).$$

Following the procedure described in Sect. "Preliminaries" on the domain $\sum { = \left[ {a,b} \right] = \left[ {0,1} \right]}$ the residual will be as

$${\mathcal{R}}\left( {\eta ,y,\tau } \right) = \frac{{\partial^{\alpha } \tilde{F}\left( {\eta ,y,\tau } \right)}}{{\partial \tau^{\alpha } }} + \frac{{\partial \tilde{F}^{2} \left( {\eta ,y,\tau } \right)}}{\partial \eta } + \frac{1}{8}\frac{{\partial^{3} \tilde{F}^{2} \left( {\eta ,y,\tau } \right)}}{{\partial \eta^{3} }} + \frac{1}{8}\frac{{\partial^{3} \tilde{F}^{2} \left( {\eta ,y,\tau } \right)}}{{\partial \eta y^{2} }}.$$

For finindg the $C_{i}$, we used the least sqarue method. Using the mathematical values of convergence control parameters from Table 2 and put in (31), we get the first order approximate solution for different values of $\alpha$ for FZK (2,2,2).

**Table 2:** Auxiliary convergence control parameters for different values of $\alpha$ for FZK(2,2,2).

$\alpha$	$C_{1}$	$C_{2}$	$C_{3}$
0.5	0.1826815783837175	0.00000056068402941548	− 0.000004025045628479711
0.75	− 0.08716270059116164	0.29147579431155546	− 0.9634327396323357
1.0	0.1819133199878481	0.000035416800066512345	− 0.0000039960642082082315

For $${{\alpha}} = 1.0$$

$$\begin{aligned} \tilde{F}\left( {\eta ,y,\tau } \right) & = \frac{4}{3}\lambda \sinh^{2} \left( {\eta + y} \right) - \frac{64}{{81}}\lambda^{4} \tau \left( {4\sinh \left( {2\eta + y} \right) - 5\sinh \left( {4\eta + y} \right)} \right) \\ &C_{1} \sinh \left( {2\eta + y} \right) + C_{2} \lambda \sinh \left( {4\eta + y} \right) + C_{3} \lambda \sinh \left( {6\eta + y} \right). \\ \end{aligned}$$

For $${{\alpha}} = 0.75$$

$$\begin{aligned} \tilde{F}\left( {\eta ,y,\tau } \right) & = \frac{4}{3}\lambda \sinh^{2} \left( {\eta + y} \right) - 0.859706\tau^{0.75} \lambda^{4} (4\sinh \left( {2\eta + y} \right) \\ & \,\,\,\, - 5\sinh \left( {4\eta + y} \right))C_{1} \sinh \left( {2\eta + y} \right) + C_{2} \lambda \sinh \left( {4\eta + y} \right) + C_{3} \lambda \sinh \left( {6\eta + y} \right). \\ \end{aligned}$$

For $${{\alpha}} = 0.5$$

$$\begin{aligned} \tilde{F}\left( {\eta ,y,\tau } \right) & = \frac{4}{3}\lambda \sinh^{2} \left( {\eta + y} \right) - 0.891559\lambda^{4} \tau^{0.5} \left( {4\sinh \left( {2\eta + y} \right) - 5\sinh \left( {4\eta + y} \right)} \right) \\ &C_{1} \sinh \left( {2\eta + y} \right) + C_{2} \lambda \sinh \left( {4\eta + y} \right) + C_{3} \lambda \sinh \left( {6\eta + y} \right). \\ \end{aligned}$$

**Figure 1:**
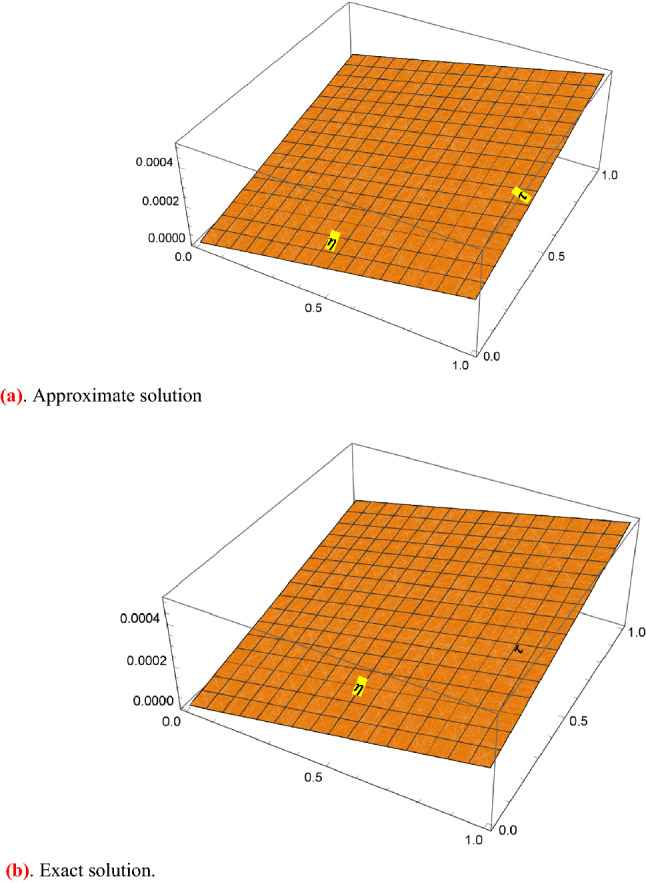
3D plots found by the suggested technique for FZK(3,3,3) at $y = 0.1$ and $\alpha = 1$.

**Figure 2:**
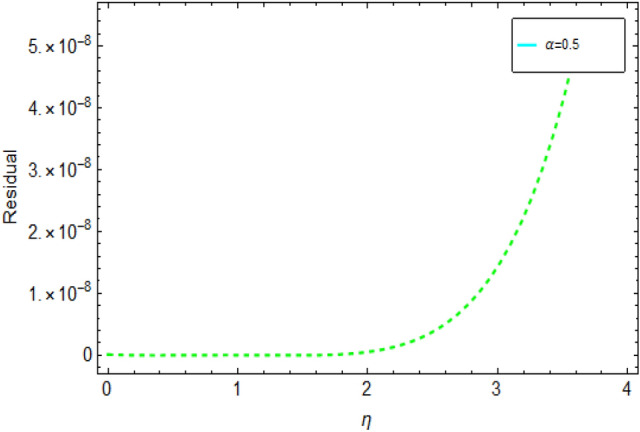
2D plots obtained by the planned process for FZK(3,3,3), Residual $\alpha = 0.5$ at $y = 0.2$ and $\tau = 0.1.$

**Figure 3:**
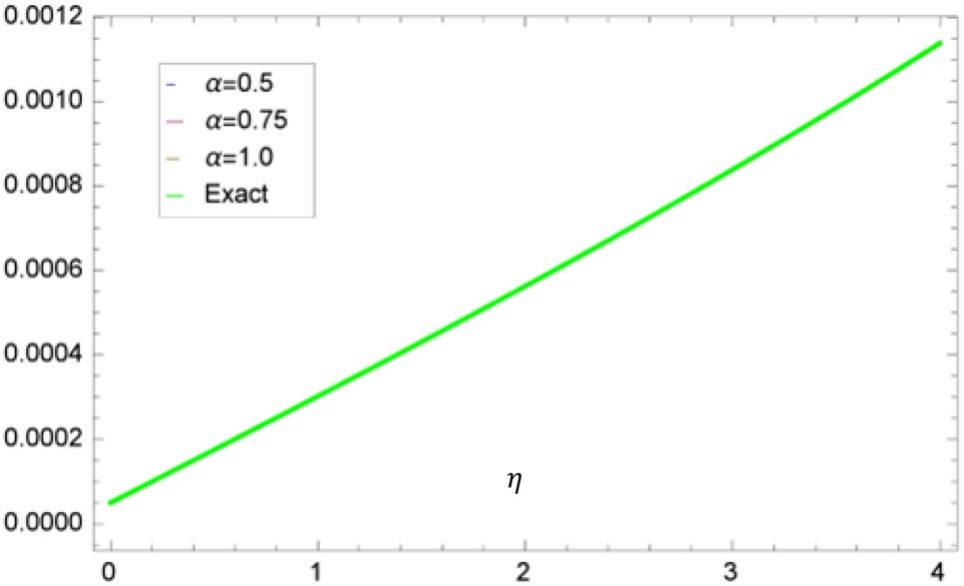
2D surfaces obtain for the approximate solution of FZK(3,3,3) for altered values of $\alpha$ when $\tau = 0.1,y = 0.2.$

**Figure 4:**
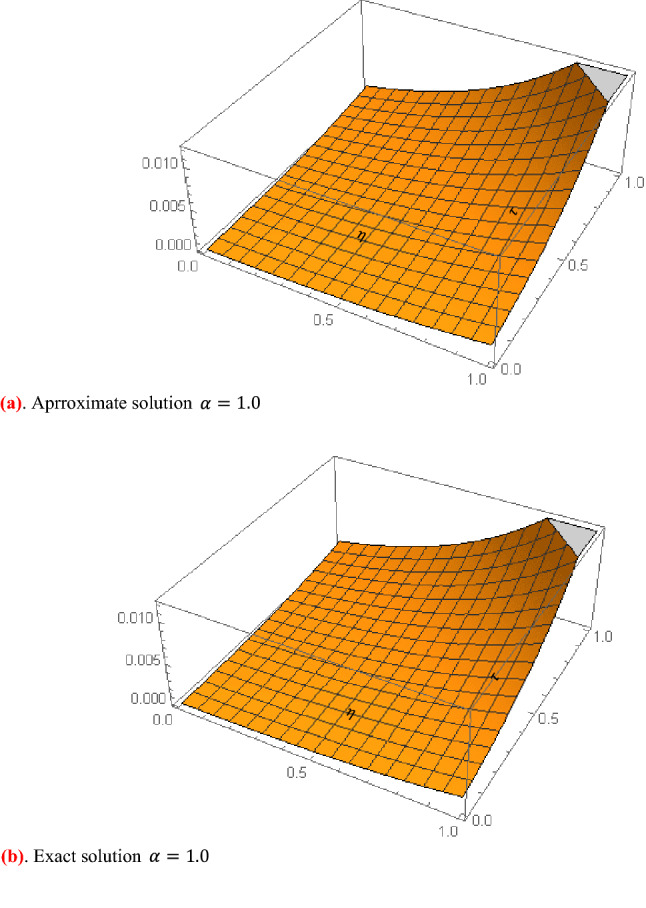
2D plots obtained by the suggested process for FZK(2,2,2) at $y = 0.2$ and $\tau = 0.1$.

**Figure 5:**
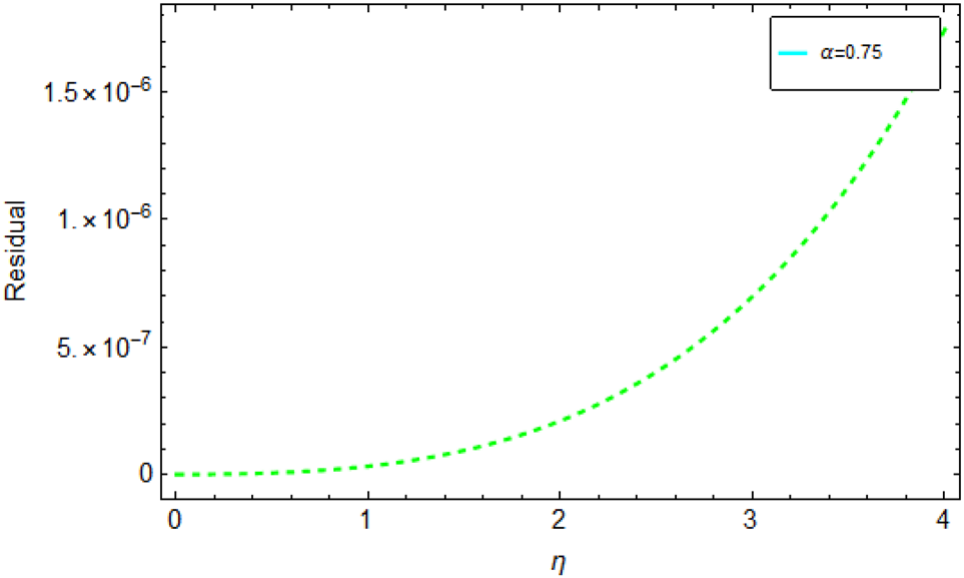
2D surface shows the Residual obtain by the projected mode for FZK(3,3,3) for dissimilar values of $\alpha$ when $\tau = 0.1,y = 0.2$.

Figure 1 shows 3D plots approximate verses exact solutions for the nonlinear fractional order FZK(3,3,3) equation when $\alpha = 1,y = 0.1$. Figure 2, displays the 2D schemes of the residual, obtained by the suggested technique for $\alpha = 0.5$ to fractional order FZK(3,3,3) equation. Figure 3, displays the 2D designs of approximate solutions obtained by the suggested mode for different values of $\alpha$ while $\tau = 0.1,y = 0.2$ to fractional order FZK(3,3,3) equation. Figure 4, shows the 3D plots obtained by the suggested process to fractional order FZK(2,2,2) equation at $\alpha = 1$ while Fig. 5 is the residual obtained by the proposed method for $\alpha = 0.75$ to fractional order FZK(2,2,2) equation.

Tables 1, 2, show the mathematical values of convergence control parameters, $C_{1} ,C_{2} ,C_{3}$ for different values of $\alpha$ for FZK(3,3,3) and FZK(2,2,2) equations. Table 3, presents the comparison of absolute errors found by the suggested technique with PIA and RPS methods for FZK(2,2,2). Similarly, Table 4, shows the absolute errors obtained by the proposed method in comparison with PIA and RPS approaches for FZK(3,3,3) equation.

**Table 3:** Comparison of absolute errors obtained by the OHAM-2 and PIA and RPS methods when $\alpha = 1.0$ and $\lambda = 0.001$ for FZK(3,3,3).

$\tau$	$\eta$	$y$	OHAM-2	Exact	Abs error VIM^28^	Abs error RPS^28^	OHAM-I^43^	Abs error OHAM-2
0.2	0.1	0.1	0.0000500092	0.0000499592	5.00091 $\times 10^{ - 5}$	3.85217 $\times 10^{ - 7}$	4.9951 $\times 10^{ - 8}$	4.9952 $\times 10^{ - 8}$
0.3	0.1	0.1	0.0000500091	0.0000499342	5.00091 $\times 10^{ - 5}$	5.75912 $\times 10^{ - 7}$	7.49279 $\times 10^{ - 8}$	7.49279 $\times 10^{ - 8}$
0.4	0.1	0.1	0.0000500091	0.0000499092	5.00091 $\times 10^{ - 5}$	7.65352 $\times 10^{ - 7}$	9.99037 $\times 10^{ - 8}$	9.99039 $\times 10^{ - 8}$
0.2	0.6	0.6	0.000302004	0.000301953	3.02003 $\times 10^{ - 4}$	4.66389 $\times 10^{ - 5}$	5.08987 $\times 10^{ - 8}$	5.09189 $\times 10^{ - 8}$
0.3	0.6	0.6	0.000302004	0.000301927	3.02003 $\times 10^{ - 4}$	6.86314 $\times 10^{ - 5}$	7.63479 $\times 10^{ - 8}$	7.63782 $\times 10^{ - 8}$
0.4	0.6	0.6	0.000302004	0.000301902	3.02003 $\times 10^{ - 4}$	8.99046 $\times 10^{ - 5}$	1.01797 $\times 10^{ - 7}$	1.01837 $\times 10^{ - 7}$
0.2	0.9	0.9	0.00045678	0.000456728	4.56780 $\times 10^{ - 4}$	5.14241 $\times 10^{ - 4}$	5.212227 $\times 10^{ - 8}$	5.21609 $\times 10^{ - 8}$
0.3	0.9	0.9	0.00045678	0.000456702	4.56780 $\times 10^{ - 4}$	7.48450 $\times 10^{ - 4}$	7.81839 $\times 10^{ - 8}$	7.82412 $\times 10^{ - 8}$
0.4	0.9	0.9	0.00045678	0.000456676	4.56780 $\times 10^{ - 4}$	9.89139 $\times 10^{ - 4}$	1.04345 $\times 10^{ - 7}$	1.04321 $\times 10^{ - 7}$

**Table 4:** Comparison of absolute errors obtained by the OHAM-2 and PIA and RPS methods when $\alpha = 1.0$ and $\lambda = 0.001$ for FZK(2,2,2).

$\tau$	$\eta$	$y$	OHAM-2	Exact	Abs error PIA^28^	Abs error RPS^28^	OHAM-I^43^	Abs error OHAM-2
0.2	0.1	0.1	0.0000540482	0.0000539388	3.85217 $\times 10^{ - 7}$	3.85217 $\times 10^{ - 7}$	2.71884 $\times 10^{ - 8}$	1.09476 $\times 10^{ - 7}$
0.3	0.1	0.1	0.0000540482	0.0000538841	5.75911 $\times 10^{ - 7}$	5.75912 $\times 10^{ - 7}$	4.07394 $\times 10^{ - 8}$	1.64171 $\times 10^{ - 7}$
0.4	0.1	0.1	0.0000540482	0.0000538294	7.65359 $\times 10^{ - 7}$	7.65352 $\times 10^{ - 7}$	5.42615 $\times 10^{ - 8}$	2.18837 $\times 10^{ - 7}$
0.2	0.6	0.6	0.00303796	0.00303651	4.66337 $\times 10^{ - 5}$	4.66389 $\times 10^{ - 5}$	6.83433 $\times 10^{ - 6}$	1.45741 $\times 10^{ - 6}$
0.3	0.6	0.6	0.00303796	0.00303578	6.86056 $\times 10^{ - 5}$	6.86314 $\times 10^{ - 5}$	1.02517 $\times 10^{ - 5}$	2.18589 $\times 10^{ - 6}$
0.4	0.6	0.6	0.00303796	0.00303505	8.98263 $\times 10^{ - 5}$	8.99046 $\times 10^{ - 5}$	1.36692 $\times 10^{ - 5}$	2.91423 $\times 10^{ - 6}$
0.2	0.9	0.9	0.0115419	0.011537	5.12131 $\times 10^{ - 4}$	5.14241 $\times 10^{ - 4}$	9.14704 $\times 10^{ - 5}$	4.87687 $\times 10^{ - 6}$
0.3	0.9	0.9	0.0115419	0.0115345	7.38186 $\times 10^{ - 4}$	7.48450 $\times 10^{ - 4}$	1.37206 $\times 10^{ - 4}$	7.31457 $\times 10^{ - 6}$
0.4	0.9	0.9	0.0115419	0.0115321	9.57942 $\times 10^{ - 4}$	9.89139 $\times 10^{ - 4}$	1.82943 $\times 10^{ - 4}$	9.75178 $\times 10^{ - 6}$

Tables 3 and 4 shows the comparison of absolute errors obtained by the OHAM-2 and PIA and RPS methods for FZK(3,3,3) and FZK(2,2,2), respectivley.

## Conclusion

The OHAM-2 methods have been applied successfully to fractional order fractional Zakharov-Kuznetsov equations. The numerical results carried out through the proposed method have been verified by 3D and 2D graphs. From the obtained results, it is clear that the fractional-order results are convergent to integer-order solutions as fractional orders are convergent to integer order. The suggested technique has a higher grade of accurateness as associated with the other approximate analytical methods. From numerical results, Nonlinear differential equations are reduced to only two linear ones. The construction of the linear operators and the auxiliary functions is done originally. We have great freedom to choose the numbers of the auxiliary functions and the optimal convergence-control parameter. The means least squares approach is used to calculate the parameter values. Our method leads to a very accurate result using only one approximation and allows us to control the convergence of the solution. We remark the construction and the properties of the linear operator L. Our procedure is effective and explicit and can be applied to any nonlinear dynamical system in the future^36–42^.

## Data Availability

All data generated or analyzed during this study are included in this published article. The datasets used and/or analysed during the current study available from the corresponding author on reasonable request.
